# Novel Allograft in the Load-Bearing Portion of the Femoral Head

**DOI:** 10.31486/toj.23.0066

**Published:** 2024

**Authors:** Andrew Renshaw, Nneoma Duru, Eric Assid, Gerard K. Williams, Misty Suri, Deryk Jones

**Affiliations:** ^1^ Ochsner Andrews Sports Medicine Institute, Ochsner Clinic Foundation, Jefferson, LA; ^2^ Boston University Chobanian & Avedisian School of Medicine, Boston, MA; ^3^ Department of Orthopedic Surgery and Rehabilitation, Howard University Hospital, Washington, DC; ^4^ The University of Queensland Medical School, Ochsner Clinical School, New Orleans, LA

**Keywords:** *Acetabuloplasty*, *allografts*, *chondrocytes*, *femur head*, *hip joint*, *osteochondral lesion*

## Abstract

**Background:** An osteochondral defect in the hip can be a painful and limiting pathologic process. The damaged joint may progress into premature osteoarthritis, further limiting a patient's functionality.

**Case Report:** A 24-year-old male presented to the clinic with left hip pain. The patient had been involved in a motor vehicle accident 3 years prior to presentation to our clinic. His injury from the high-speed accident required intramedullary rod fixation for a right-sided (contralateral) subtrochanteric femur fracture. The patient complained of left groin pain when in a sitting position, with activities of daily living, and with exercise. He failed conservative management consisting of nonsteroidal anti-inflammatory drugs and physical therapy. Imaging on presentation demonstrated an osteochondral defect in the weight-bearing portion of the left femoral head consistent with an International Cartilage Repair Society grade 4b lesion, a cam lesion was noted on assessment of bone morphology, and magnetic resonance imaging revealed degenerative labral pathology. The patient was treated with surgical hip dislocation through a modified Hardinge approach, femoral head osteochondral allograft transplantation using a Missouri Osteochondral Preservation System (MOPS) graft, acetabuloplasty, femoral neck osteoplasty, and open labral repair.

**Conclusion:** Femoral head osteochondral MOPS allograft transplantation is a viable technique for joint preservation in young patients with posttraumatic osteochondral defects of the femoral head.

## INTRODUCTION

An osteochondral defect is a focal area of damaged articular cartilage and subchondral bone within a joint. These lesions can be caused by biomechanical macrotraumatic or microtraumatic factors, avascular necrosis, osteochondritis dissecans, infection, inflammatory mediators and conditions, and genetic predisposition.^1^ Symptoms vary widely; patients can be asymptomatic or they can experience pain, as well as locking and catching sensations leading to functional impairment.^2^ Regardless of symptomatology, the presence of osteochondral damage alone may progress with further cartilage loss, release of inflammatory mediators, and eventual osteoarthritis—even in healthy adults.^3^ Development of atraumatic osteoarthritis or secondary posttraumatic arthritis in young patients is troublesome for treating physicians, as definitive treatment often requires joint replacement;^4^ however, in young patients with full life expectancy and ambitions to return to normal activities, the lifespan of the arthroplasty implant often is less than that of the patient.^4-6^ Joint preservation techniques are appealing to delay or prevent arthritic progression.^7-13^

No standard surgical options have been established for treatment of osteochondral defects of the femoral head, but options such as microfracture, autologous chondrocyte implantation, osteochondral autograft transfer or mosaicplasty, and osteochondral allograft transplantation have been adapted from the knee literature.^7-13^ While potential drawbacks for osteochondral autograft or allograft exist—donor site morbidity and immunogenic bone, respectively—both techniques have the advantage of restoring the subchondral bone scaffold while providing mature, immunoprivileged cartilage with viable chondrocytes and extracellular matrix.^14-18^ Reports in the literature of femoral head osteochondral allograft transplantation for various pathologic conditions demonstrate favorable outcomes at short-term to midterm follow-up.^19,20^

Osteochondral allograft transplantation success is largely dependent on chondrocyte viability and extracellular matrix composition, with chondrocyte viability decreasing over time from harvest.^21,22^ Williams et al demonstrated a dramatic decrease in viability over time: at 15 days, 80.2% of chondrocytes were viable, while at 45 days, 64.6% of chondrocytes remained viable.^23^ Mandatory disease screening protocols take approximately 2 weeks to complete before osteochondral allografts can be distributed for clinical use, making the window for surgical implantation narrow and limiting allograft clinical use while resulting in financial losses and waste of donor tissue.^24^

The Missouri Osteochondral Preservation System (MOPS) is a novel medium designed to increase the viability of donor tissue cells beyond the typical 14-day period of fresh graft storage (4 °C) medium that is the standard of care for osteochondral allografts.^25^ MOPS grafts have demonstrated increased chondrocyte viability for up to 60 days before levels become suboptimal for implantation.^24^ The increased numbers of viable cells in the donor grafts have potential benefits for graft survival and incorporation rates at the site of implantation.^22^ Buyuk et al showed promising functional outcomes for defects of the knee treated with osteochondral allografts stored using the MOPS protocol.^26^

To our knowledge, no existing literature describes MOPS osteochondral allografts being used to treat femoral head cartilage defects. This case demonstrates the use of a MOPS femoral head osteochondral allograft for a large, symptomatic osteochondral defect in a patient who had sustained improvements in functional outcomes scores more than 5 years postoperatively.

## CASE REPORT

A 24-year-old male presented to the clinic with long-term left hip pain secondary to a high-speed motor vehicle accident 3 years prior that required surgical intervention to his contralateral hip. He was a restrained driver. He sustained multiple injuries, including a right subtrochanteric femur fracture treated with intramedullary nailing. After his initial rehabilitation period status post intramedullary nailing, the patient's hip pain slowly progressed. The patient explored treatment once he began to experience stiffness and increased functional debilitation. Initial treatment encompassed pain management with nonsteroidal anti-inflammatory drugs and physical rehabilitation. He continued to have pain and stiffness in his left hip and groin despite more than 6 weeks of conservative treatment. Preoperative physical examination revealed that he flexed his hip to 110°, externally rotated to 30°, and internally rotated to 10° with pain. Flexion, adduction, and internal rotation; flexion, abduction, and external rotation; and log roll test were positive, demonstrating significant asymmetry compared to the opposite hip. Bridge test was also positive.

**Figure 1.**
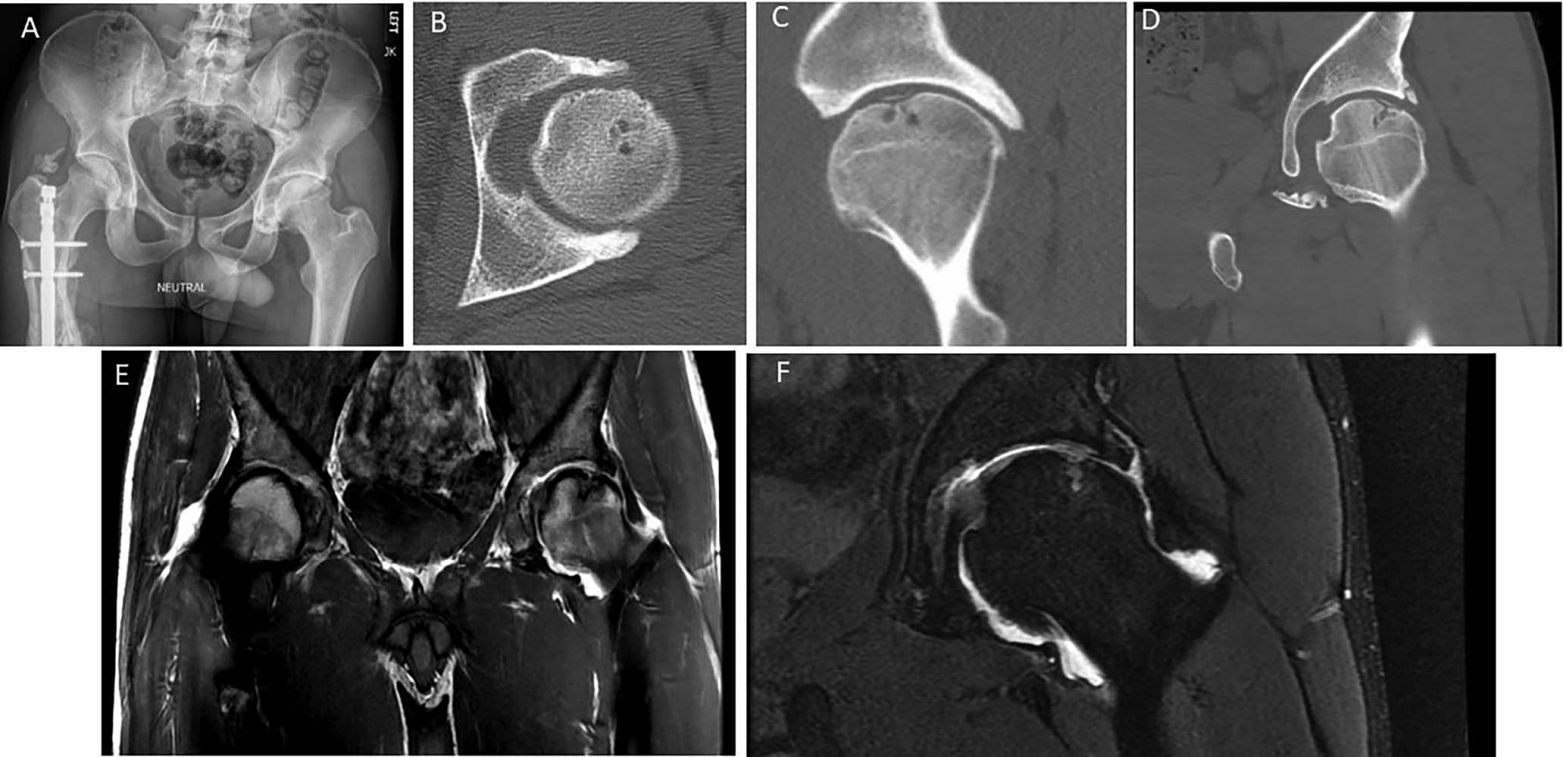
Preoperative imaging demonstrates hip pathology. (A) Anterior/posterior bilateral hip radiograph shows cam morphology and subchondral sclerosis femoral head on the left and intramedullary nail on the right. (B) Computed tomography (CT) axial series shows calcified labrum, subchondral cysts, and osteophytes of the left hip. (C) CT sagittal series of the left hip. (D) CT coronal series of the left hip. (E) Magnetic resonance imaging (MRI) T1 coronal series of the bilateral hips demonstrates labral tear, joint space narrowing, and a cam lesion. (F) MRI T2 coronal series proton density fast spin of the left hip demonstrates a labral tear, subchondral edema, joint space narrowing, and a cam lesion.

Initial bilateral hip anterior/posterior radiographs demonstrated an osteochondral defect of the weight-bearing portion of the left femoral head, cam and pincer lesions, subchondral sclerosis, and early joint space narrowing in the left femoroacetabular joint (Figure 1A). Preoperative computed tomography (CT) imaging demonstrated labral calcifications, subchondral cysts, and further evidence of degenerative joint disease (Figures 1B, 1C, and 1D). Magnetic resonance imaging (MRI) demonstrated evidence of labral tearing and the cam lesion of the femoral neck (Figures 1E and 1F).

**Figure 2.**
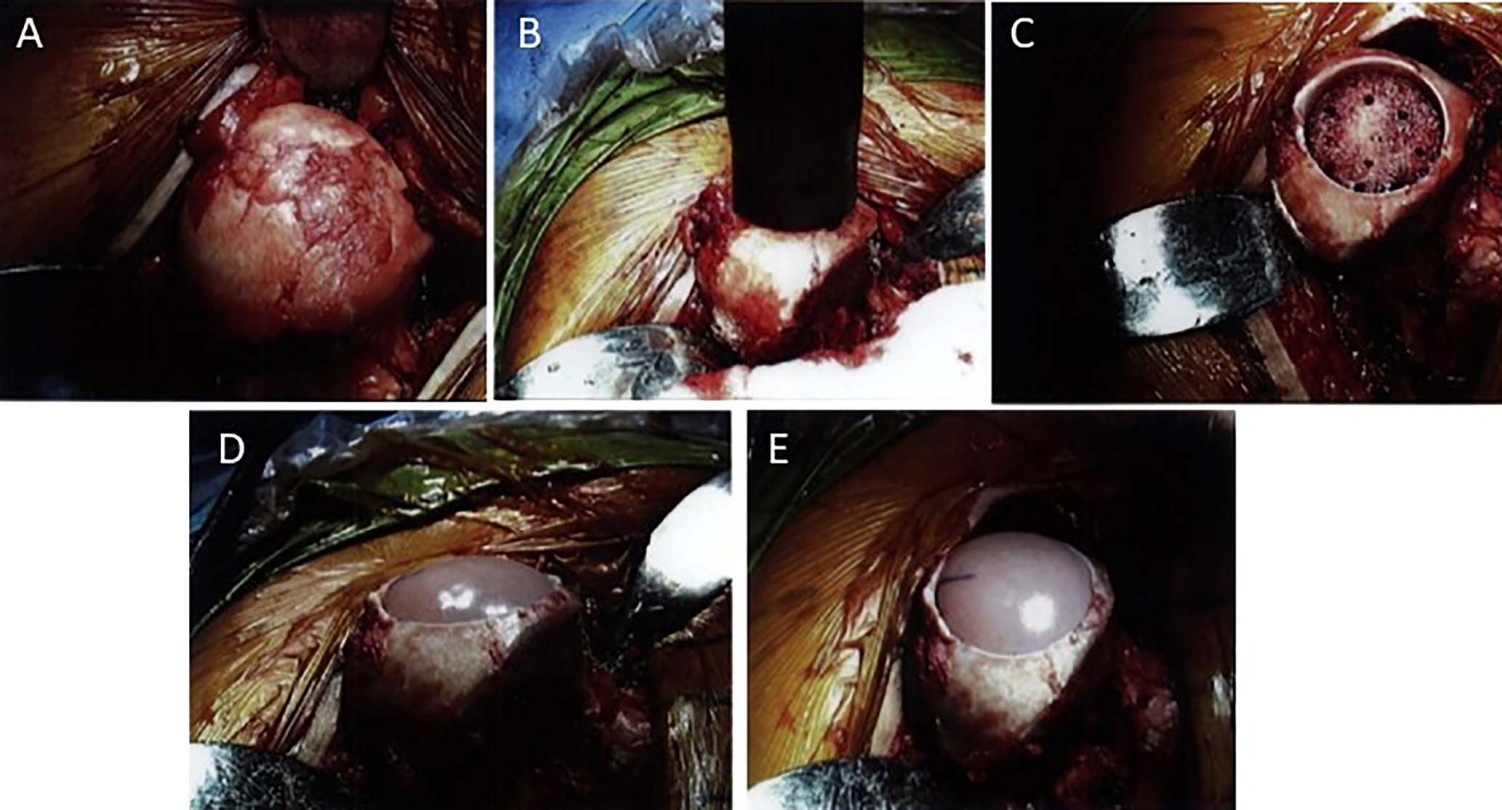
Intraoperative surgical pictures depict the surgical hip dislocation, subsequent reaming, and graft transplantation. (A) Open approach to the left hip shows the International Cartilage Repair Society grade 4b lesion on the anterior superior surface of the femoral head. (B) Central reamer over the entirety of the cartilage lesion. (C) Open view of the left hip shows reamed surface of the left femoral head. (D and E) Open views show Missouri Osteochondral Preservation System osteochondral allograft transplanted into the left femoral head.

Given the patient's young age and desire to return to normal activities of daily living, a joint-preserving surgical intervention was chosen. Operative treatment included an anterolateral Hardinge approach^27^ with minimal trochanteric osteotomy allowing anterior left hip dislocation. Open labral repair, acetabuloplasty, femoral neck osteoplasty, and MOPS osteochondral allograft transplantation to the femoral head were performed. A 35-mm diameter osteochondral dowel graft was implanted, restoring the contour of healthy femoral head cartilage (Figure 2). The trochanteric osteotomy was repaired with transosseous suture fixation using three #2 ORTHOCORD sutures (DePuy Synthes) placed through 2.0-mm drill holes. The patient was maintained at toe-touch to 25% partial weight-bearing with a hip abduction brace for 4 weeks and was advanced to 50% partial weight-bearing at 4 to 6 weeks and full weight-bearing as tolerated at 6 to 8 weeks. Range of motion was full extension to 60° flexion for 2 weeks, followed by 90° flexion from 2 to 4 weeks. Internal and external rotation and active abduction were limited for 6 weeks. The patient was advanced to full weight-bearing in an Össur hip unloader brace (Össur hf) at 6 weeks with instructions to wear the brace at all times until 3 months. The labral repair was protected per standard protocol for 6 months following surgery. The patient was advised to use an EXOGEN bone stimulator (Bioventus Inc) twice daily for 30 minutes to augment osseous integration of the allograft.

Preoperatively, the patient's visual analog scale (VAS) score was 8/10, with pain awakening him 2 nights of the week. At his 2-week postoperative visit, the patient reported 8/10 pain, but the pain no longer awakened him at night. At 6 weeks, as weight-bearing and range of motion were advanced, the patient's VAS score increased to 9/10, with pain awakening him 2 nights of the week. A core strengthening program was instituted at 6 weeks. At 3 months, his VAS score decreased to 6/10 with activities and 2/10 at rest, and the patient was no longer awakening at night because of pain. Hip flexion was approximately 100°, allowing the patient to sit in a chair and enter a vehicle comfortably. Strength at the 3-month follow-up was 5/5 hip flexion, abduction, and extension. At 6 months, the patient reported no pain, he was able to perform normal activities of daily living, and he was actively involved in school without difficulty regarding his left hip. His hip flexion was 110° (symmetric to his right hip). Left hip internal rotation was 15° and external rotation was 45° compared to right hip internal rotation of 0° and external rotation of 30°.

**Figure 3.**
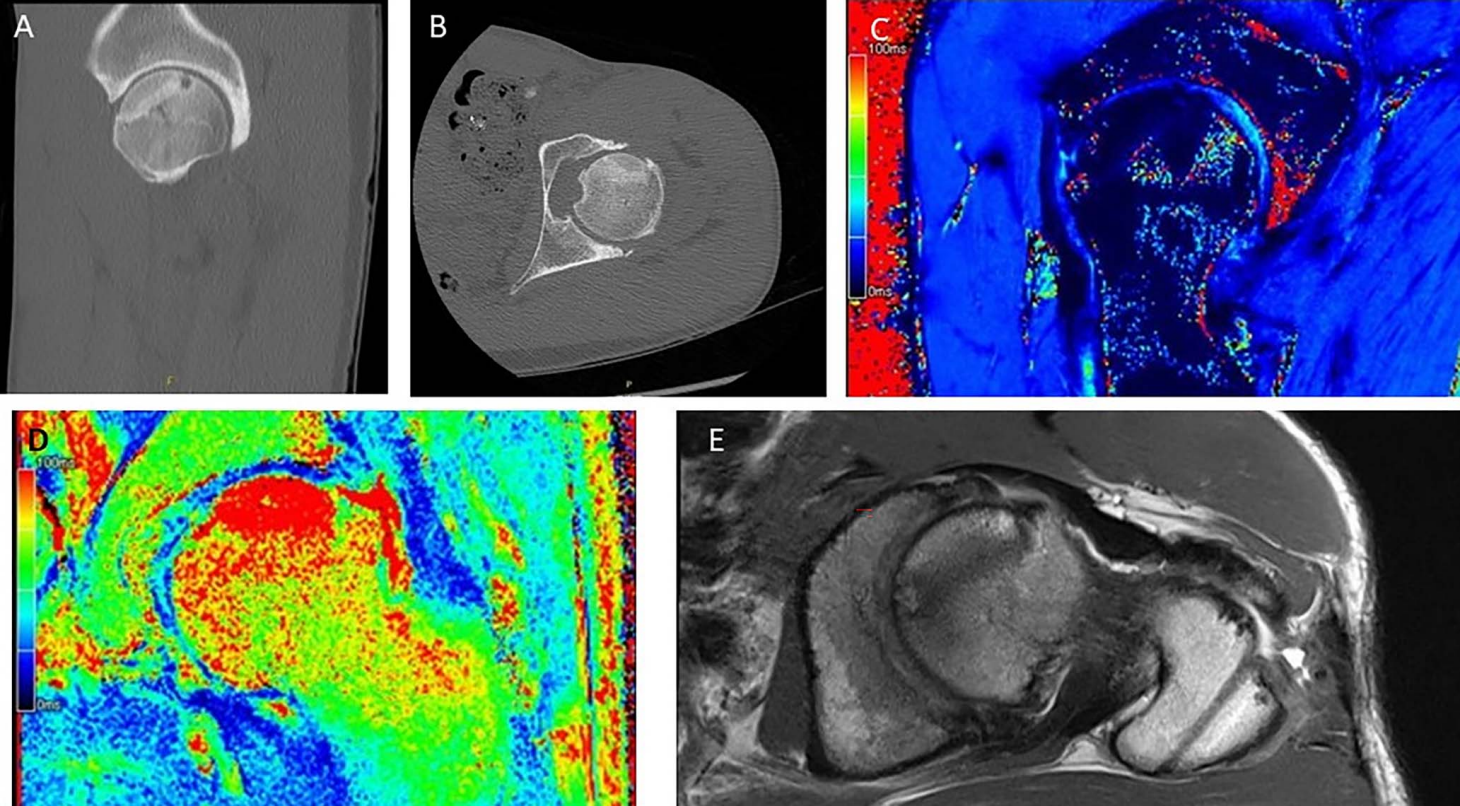
Postoperative imaging. (A) Computed tomography (CT) sagittal series demonstrates joint congruity and osseous integration of the left hip. (B) CT axial series demonstrates joint congruity and osseous integration of the left hip. (C and D) T2 magnetic resonance imaging (MRI) with cartilage mapping shows chondrocyte viability of the left hip. (E) MRI axial-oblique series demonstrates joint congruity and osseous integration of the left hip.

Repeat CT and MRI scans performed at 7 months demonstrated excellent osseous integration of the MOPS allograft with delamination at the cartilage layer and maintained congruency with the native femoral head articular cartilage (Figure 3).

**Figure 4.**
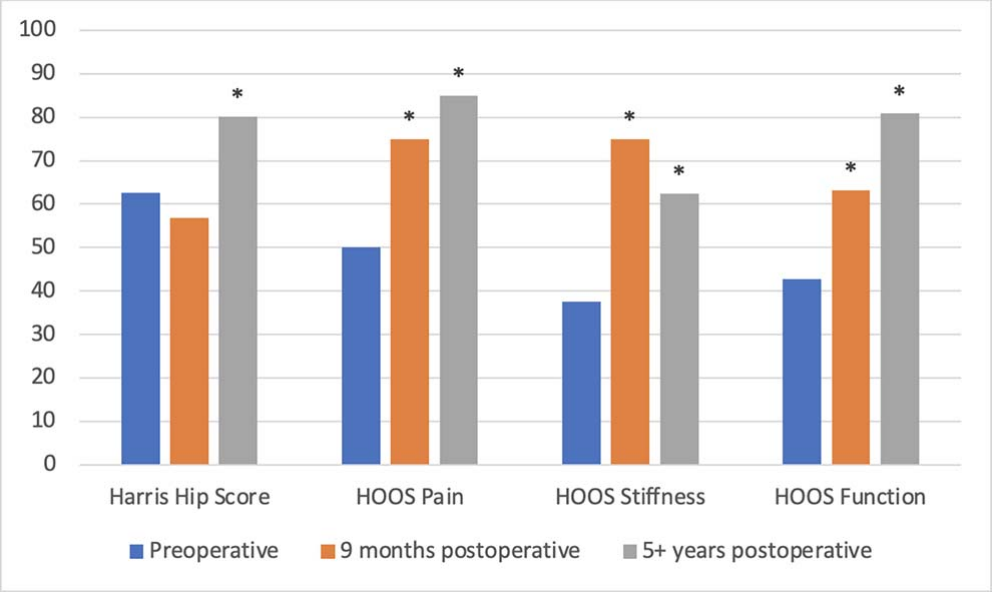
Modified Harris Hip Score and hip disability and osteoarthritis outcome scores (HOOS) at preoperative and postoperative time points. The asterisks indicate significance of the minimal clinically important difference. For all scales, higher scores indicate better outcomes.

Functional scores demonstrated improvement from baseline to 60+ months with an 18-point improvement in modified Harris Hip Score (mHHS), a 35-point improvement in hip disability and osteoarthritis outcome score (HOOS) pain, a 25-point improvement in HOOS stiffness, and a 38-point improvement in HOOS function (Table^28^ and Figure 4).

**Table.** Patient-Reported Outcome Measures Scores at Baseline and Postoperatively

	Outcome Measure			
Time Point	Harris Hip Score	HOOS Pain	HOOS Stiffness	HOOS Function
Preoperative (baseline)	62.64	50	37.5	42.65
9 months postoperative	56.9	75^a^	75.0^a^	63.25^a^
5+ years postoperative	80.22^a^	85^a^	62.5^a^	80.88^a^

^a^Significance of the minimal clinically important difference.^28^

Notes: The modified Harris Hip Score and the hip disability and osteoarthritis outcome score (HOOS) were used for these assessments. For all scales, higher scores indicate better outcomes.

## DISCUSSION

Osteochondral defects of the femoral head in young, active patients can pose significant treatment challenges for clinicians. The poor healing potential of articular cartilage and high forces across the hip joint with daily and high-impact activities can lead to progression of arthritic disease of the femoral head.^29,30^ Total hip arthroplasty in young, active patients can lead to accelerated wear and eventual failure, despite newer iterations of polyethylene that demonstrate improved wear characteristics.^31,32^ Several small studies report favorable short-term outcomes after microfracture of the hip in lieu of arthroplasty.^8-13,33^ Larger studies with longer follow-up are needed to better assess the effectiveness of microfracture techniques in the hip.

Fontana and de Girolamo treated acetabular chondral lesions with concomitant arthroscopic correction of femoral acetabular impingement.^34^ In their randomized controlled trial, they compared 77 patients treated with microfracture to 70 patients treated with autologous matrix-induced chondrogenesis (AMIC), a technique that combined microfracture with arthroscopic placement of a resorbable type I/III matrix (Chondro-Gide). In the Fontana and de Girolamo study, AMIC was statistically superior to isolated microfracture. Six patients who underwent microfracture treatment had total hip arthroplasty at 5 years compared to no patients who underwent AMIC. Functional deterioration was noted in the microfracture arm 2 years postoperatively.^34^

The use of mosaicplasty in traumatic femoral head lesions has been reported, with the typical harvest from the superolateral trochlea of the knee.^35-37^ Won et al harvested four 4.5-mm plugs from the minimal weight-bearing cartilaginous region of the femoral head, an area located at the cam lesion in typical femoral acetabular impingement conditions.^38^ In these reports, plugs were used to fill in persistent defects of the femoral head after repair of the traumatic osteocartilaginous fragments with chondral darts. Short-term results were good, but the technique had size limitations.

Fontana and colleagues compared 15 patients treated with arthroscopic debridement of femoral head lesions to 15 patients treated with a 2-staged matrix-induced autologous chondrocyte implantation (MACI) procedure using BioSeed-C, a resorbable polymer-based scaffold (2 × 3 cm × 0.2 cm height) of polyglycolic/polylactic acid and polydioxanone.^39^ Cells were harvested from the acetabular pulvinar region following arthroscopic evaluation of the defect site. Mean lesion size was 2.6 cm^2^, and the mean HHS was similar in both groups at baseline, but an average 39.1-point improvement in HHS was seen in the MACI arm vs a 10.3-point improvement in HHS in the debridement arm at 74-month follow-up.

Arthroscopic application of NOVOCART Inject (a combination of autologous cartilage cells and an in situ polymerizable hydrogel) through a deformable applicator has been compared with implantation of Chondrosphere, created by combining patient serum with autologous chondrocytes in hydrogel-coated plates.^12,40^ In the Thier et al study, a total of 29 patients were available for final follow-up with demonstrated improvements in Euro-Quol group score, International Hip Outcome Tool, Non-Arthritic Hip Score, and Short-Form Health Survey in both groups, with greater statistically significant improvements in the NOVOCART patients. Interestingly, all lesions were on the acetabulum.^12^

Osteochondral allograft transplantation has been used to treat larger lesions for some time with positive results, in contrast to smaller lesions which have been managed with mosaicplasty, microfracture, and autologous chondrocyte transplantation.^41^ Moreau et al reported 8-year follow-up after using a 30-mm fresh osteochondral allograft in a 17-year-old patient with a chondroblastoma.^42^ Mei et al reported transplantation of fresh femoral head allografts in 22 patients within 2 weeks of donation.^19^ As in the Moreau et al chondroblastoma case,^42^ all 22 patients in the Mei et al study were approached through a trochanteric slide anterolaterally, with posttransplantation fixation of the osteotomy with screws.^19^ At 68.8 months postoperatively, patients demonstrated a mean improvement of 28.5 points in the mHHS. Five patients were converted to total hip arthroplasty with documented radiographic progression by Kellgren and Lawrence assessment in 4 patients. Kaplan-Meier analysis of graft survivorship was 86.4% ± 7.3% at 2 years, 78.5% ± 10.0% at 5 years, and 67.3% ± 13.5% at 9 years.^19^

We used a modified Hardinge approach in our surgery,^27^ through the intermuscular plane between the anterior two-third and posterior one-third of the gluteus medius muscle, followed by dissection through the gluteus minimus muscle and capsule to expose the femoral head. As in other anterolateral approaches, the hip is dislocated anteriorly, maintaining vascularity to the femoral head, but this approach minimizes trochanteric morbidity associated with other lateral-based exposures.^43-46^ We created a minimal trochanteric sleeve in our approach to limit morbidity while allowing bone healing following transosseous repair with #2 diameter sutures.

Another unique feature of our procedure was the use of the MOPS femoral head graft. Osteochondral allograft transplantation has demonstrated prolonged benefits in distal femoral chondral pathology using grafts transplanted within 2 weeks of donation.^47,48^ LaPrade et al demonstrated similar improvements in femoral condylar lesions using refrigerated (4 °C) or standard preserved grafts.^18^ As previously discussed, refrigerated grafts represent slowly decaying implants with limited cell viability after 29 to 45 days.^23^ MOPS grafts have demonstrated better outcomes than standard preserved grafts in the short-term follow-up of patellofemoral conditions treated with osteochondral allograft transplantation.^26^ MOPS grafts have the potential to impact outcomes at intermediate to long-term follow-up as chondrocyte viability at implantation is higher at longer time points from donation to implantation. The practical benefit of MOPS osteochondral allografts is that surgeons and patients have more time to schedule and implant the graft without concern for chondrocyte viability or integrity.

To our knowledge, our case is the first report of femoral head osteochondral allograft transplantation with use of a MOPS femoral head graft, and the patient had excellent outcomes. Further studies comparing MOPS grafts with standard preserved grafts are warranted. This case demonstrates an opportunity for expanding and improving upon the existing options for treating hip osteochondral lesions through the use of the MOPS protocol.

## CONCLUSION

Femoral head osteochondral MOPS allograft transplantation is a viable technique for joint preservation in young patients with posttraumatic osteochondral defects of the femoral head.

## Notes

*This article meets the Accreditation Council for Graduate Medical Education and the American Board of Medical Specialties Maintenance of Certification competencies for Patient Care and Medical Knowledge.*
